# Dementia and aging populations—A global priority for contextualized research and health policy

**DOI:** 10.1371/journal.pmed.1002275

**Published:** 2017-03-28

**Authors:** Carol Brayne, Bruce Miller

**Affiliations:** 1 Cambridge Institute of Public Health, Cambridge University, Cambridge, United Kingdom; 2 Aging and Memory Center, University of California San Francisco, San Francisco, California, United States of America

## Abstract

In this month’s Editorial, Guest Editors Carol Brayne and Bruce Miller discuss research and commentary published in March and future directions for dementia research.

Current evidence suggests substantial increases in dementia numbers across the world [1], although there is evidence in some countries of age for age reductions in both prevalence and incidence across time [2]. Thinking about future directions for dementia research, it is important to incorporate our understanding of global challenges for contemporary and future populations. We know that a generalized phenomenon of population aging is occurring across the globe, and that in low- and middle-income countries, many of the diseases associated with affluence are rising in incidence, identified by both the UN and WHO as concerns. These population changes do not affect all people equally. For example, health conditions that are risk factors for dementia are both rising (diabetes) and falling (stroke) in high-income countries. The implications for dementia outcomes for populations cannot be assumed as, for example, the complications of diabetes are declining in some populations and differ by population, inequality, and generation [3]. In particular, people who are disadvantaged socioeconomically have lives that are shorter than those of richer population groups and are even so at higher risk for dementia [3]—such groups have not, to date, been well served by the major investments in dementia research.

There is increasing realization that public health measures will play an important role in the drive to protect world populations from cognitive impairment and dementia, but there is relatively little research in this area, acknowledgement of lack of knowledge (e.g., recent road map) and a wide variability in actions taken to address these in different societies and certainly in their effectiveness. In the public health domain, this special issue of *PLOS Medicine* explores potential for addressing reversible factors in varied cultural and socioeconomic environments. Not surprisingly, a “one size fits all” approach does not appear to work. In parallel, research studies published in this special issue also help to advance understanding of genetics and dementia, emphasizing a heterogeneity of risk factors for different individuals and populations.

Genetics play a critical role in defining risk for neurodegenerative disorders, particularly for those at familial risk, while helping to select targets for therapies. The systematic study of Alzheimer disease (AD) began seriously in the mid-1980s with the discovery that the major protein in plaques consisted of a 42-amino-acid protein, amyloid-β-42 [4]. Subsequently, discovery of genetic mutations in the amyloid precursor protein genes [5], presenilin 1 [6] and presenilin 2 [7], as causes for familial AD supported an amyloid hypothesis for AD, as all of these mutations influenced the production and metabolism of amyloid-β-42. Yet, these mutations are rare in most populations, accounting for less than 2% of AD cases. More than 30 years after these seminal discoveries, nearly all of the investments of the pharmaceutical industry have been devoted to either decreasing the production of amyloid-β-42 or increasing its clearance. While the jury is still out on the amyloid hypothesis, the findings of many unsuccessful studies have led investigators to consider other potential targets for AD. Further research themes have emerged around frontotemporal dementia and Parkinson disease dementia with tau, progranulin C9orf72, α-synuclein, and LRRK2 all having been shown to play roles in these disorders. The discovery of different genetic factors associated with distinct conditions has encouraged more diverse approaches.

Genetics has evolved from the study of families to the use of large genetic databases to explore genes conferring lower, but potentially clinically relevant, absolute risks for dementia. This approach is reinforced and expanded upon in this special issue, with investigations of multiple genes contributing to cognitive decline and dementia. In a study of 17,008 AD cases and 37,154 controls, Desikan and colleagues computed a polygenic risk score that was highly predictive for age of onset of individuals for dementia [8]. Yokoyama and colleagues note a genetic contribution to AD through inflammatory pathways in a study of HLA markers [9]. They report a role of the class I haplotype A*03:01~B*07:02 and class II haplotype DRB1*15:01- DQA1*01:02- DQB1*06:02 (DR15) as risk factors for AD. The inflammatory risk appeared particularly important in populations that did not carry the apolipoprotein ε4 allele, which is known to confer increased risk of AD. By contrast, Lipnicki and colleagues report on harmonized longitudinal data for 14 cohorts from 12 countries [10]: different rates of cognitive decline were seen for those of different ethnicity, for men versus women, and for apolipoprotein ε4 carriers. In work from Blacker and colleagues, the slow conversion rate of patients with mild cognitive impairment in aging cohorts (except for ε4 carriers) is described, with the study suggesting a need for treatment trials that are enriched for participants carrying ε4 [11]. Finally, Campion and colleagues describe a French cohort in which polymorphisms and known mutations in APP, PSEN1, and PSEN2 were found in a population with early-onset AD, including sporadic cases [12].

Other papers published in this special issue reflect a wider range of indicative areas in which future research should be more strongly focused. These are green shoots where, to date, there has not been sufficient work to keep up with changes in global populations. These include new methodologies such as analysis of activities of daily living for diagnosis of cognitive impairment and dementia [13] and ambitious approaches to designing systems for improved care and support of people with dementia in a pilot study [14]. Other contributions include findings and commentary on dementia phenotypes in low- and middle-income countries [15,16], the importance of the dementia syndrome and severe cognitive impairment and their underlying neurobiology in the oldest old [17], social and behavioral science approaches to life course risk [18], the role of head injury as an example of a risk that varies widely across the world [19], the rising appreciation of the role of cognitive or brain reserve in protecting individuals from the expression of dementia in the face of brain changes related to aging and active neuropathology [20], and the applications for, as well as challenges in the interpretation of, routine data [21]. In addition, we need greater attention to the cultural framing of dementia [22], which has changed so much in so many parts of the world, with pressure towards diagnosis without symptoms. Given that the reason for all relevant research is the societal burden experienced by aging populations, not the neurobiological and neuropathological expression of intermediate states, this cultural and societal aspect is very important.

As science improves our understanding of dementia as well as its relationship to ageing in different populations, better prediction and prevention will be greatly facilitated by studies such as those reported in this month’s *PLOS Medicine* issue, with progress being made around public health and the genetic and behavioral risks for dementia. However, it would be possible to spend the world’s entire research budget for dementia research on elegant science with limited reproducibility and even less generalizability, thereby failing to make any difference to the world’s individuals, families, and societies struggling with an increasing burden of dementia. Resources are finite, with energy, materials, and the environment all under pressure from the increasing global population, at least partly due to the aging phenomenon. Research focused on dementia and aging research also needs to look at the longer-term implications of the types of research that are being conducted—to assess whether the models we are pursuing will contribute to, or mitigate against, the challenges that we as societies face. For the benefit of future generations, governments and research funders should work towards an era in which the age-adjusted risk for dementia might be lower, inequalities reduced, and the human population more likely to live long and sustainable lives, and live them well, as a result of judicious and forward thinking research.
